# New analogs of 5-substituted-2(1*H*)-pyridone containing of natural amino acids as potential drugs in idiopathic pulmonary fibrosis. Investigation *in silico* and preliminary *in vitro*

**DOI:** 10.3389/abp.2025.15218

**Published:** 2025-11-10

**Authors:** Krystyna Dzierzbicka, Maria Skrzypkowska, Monika Gensicka-Kowalewska, Mateusz Daśko, Bartosz Słomiński

**Affiliations:** 1 Department of Organic Chemistry, Faculty of Chemistry, Gdansk University of Technology, Gdansk, Poland; 2 Department of Medical Immunology, Medical University of Gdansk, Gdansk, Poland; 3 Department of Inorganic Chemistry, Faculty of Chemistry, Gdansk University of Technology, Gdansk, Poland

**Keywords:** pirfenidone, PFD, amino acids, idiopathic pulmonary fibrosis, IPF

## Abstract

The aim of our work was to analyze new functionalized analogues of 5-substituted-2(1*H*)-pyridone containing of natural amino acids derivatives as a potential drugs in idiopathic pulmonary fibrosis (IPF). The creation of connections with natural amino acids was aimed at obtaining anti-fibrotic compounds with better water solubility, increased hydrophilicity, lower toxicity and better pharmacokinetic properties. For the docking studies the corresponding grid box parameters were used: PARPγ, ALK5 andp38. During our initial research we have synthesized and performed biological *in vitro* studies for two analogues selected on the basis of molecular modeling: **6b** and **6f**. MTT test have been performed to select concentrations of PFD derivatives for subsequent analysis. We have analyzed HLA-DR and CXCR4 expression on fibroblasts and 24 h migration of TGF-β1-stimulated fibroblasts. We have also explored proliferation and production of TGF-β1 as well as IL-17 by CD3/CD28 beads-stimulated PBMCs. Preliminary studies show that the designed compounds exhibit promising potential as anti-fibrotic therapeutics. Therefore, their activity is worth further exploring.

## Introduction

Idiopathic pulmonary fibrosis (IPF) is a chronic interstitial pneumonia with very unfavorable prognosis (Harari and Caminati, 2015; Sdhah et al., 2021). It is characterized by the excessive and progressive deposition of extracellular matrix that destroys lung tissue and fatally deteriorates organ function. The median survival of the sufferers has been estimated at 2.5–5 years after the diagnosis (Sakai and Tager, 2013; Bonham et al., 2019). IPF is considered rare condition that usually affects older adults. The disease is acknowledged as a consequence of compromised activity of repetitively injured alveolar epithelial cells and their impaired interactions with fibroblasts that lead to induction and/or recruitment of 3^rd^ population, so called myofibroblasts. Myofibroblasts are a direct source of excessive extracellular matrix components that disturb gas exchange between the alveoli and blood vessels (Richeldi et al., 2017; Shenderov et al., 2021). Numerous environmental and genetic risk factors have also been implicated in IPF pathogenesis, including exposure to infections, cigarette smoke or occupational dust (Richeldi et al., 2017). Within genetic factors sequences connected with: host defense, epithelial barriers, telomere structure maintenance or secretory activity of epithelial cells have been recognized (Garcia, 2018). So far, the only effective disease-modifying therapies for IPF comprise of tyrosine kinase inhibitor - nintedanib and a pyridine–pirfenidone (PFD). Nintedanib blocks various fibrosis-related receptors, including: fibroblast growth factor receptor (FGFR), platelet-derived growth factor receptor (PDGFR) and vascular endothelial growth factor receptor (VEGFR) (Cerri et al., 2019). Exact mechanisms of pirfenidone’s action remain to be described (Richeldi et al., 2017). As their introduction completely changed IPF’s clinical management, their use could not be overestimated (Seifirad, 2020). Unfortunately, both therapeutics merely slow down disease progression and are often not well tolerated (Shenderov et al., 2021). PFD (5-methyl-1-phenyl-2-(1*H*)-pyridone) is being recognized for its numerous beneficial effects–e.g., it reduces the decline in the forced vital capacity (FVC) of the lungs, reduces exacerbations as well as limits hospitalization demands for respiratory failure. However, it requires high doses administration and is known to often cause significant side effects (Sakai and Tager, 2013; Bonham et al., 2019), including gastrointestinal disturbances and fatigue what indicates that PFD is not well tolerated in many patients who require long-term treatment (Han et al., 2019).

The ultimate IPF’s treatment still requires lung transplant, with only fraction of patients undergoing the procedure and only approximately half of them surviving 5 years (Lederer and Martinez, 2018). Therefore, regardless of advances made in IPF therapy, there is still considerable room for improvement and need to obtain molecules with enhanced properties and less detrimental side effect (Parimon et al., 2020).

The aim of our research was to obtain antifibrotic compounds with better water solubility, increased hydrophilicity, lower toxicity and better pharmacokinetic properties as a possible future PFD replacement.

## Materials and methods

### Computational studies

#### Ligands preparation for molecular docking

The 3D structure of ligands **6a-6m** (Supplementary Table S1) were prepared with the Portable HyperChem 8.0.7 Release (Hypercube, Inc., Gainesville, FL, USA). Prior to docking calculations, the structure of each ligand was optimized using a MM+ force field and the Polak–Ribière conjugate gradient algorithm (terminating at a gradient of 0.05 kcal mol^−1^ Å^−1^).

#### Protein preparation for molecular docking

The X-ray structures of the PARPγ, ALK5, and p38 used for molecular modeling studies were taken from the Protein Databank (Protein Data Bank accession codes: 2PRG, 1RW8, and 1KV2, respectively). After standard preparation procedure (including removal of water molecules and other ligands as well as addition hydrogen atoms and Gasteiger charges to each atom) docking analysis was carried out. In the case of PARPγ, the amino acid chains B and C were additionally removed from the structure 2PRG.

#### Molecular docking

Docking studies were carried out using Autodock Vina 1.1.2 software (The Molecular Graphic Laboratory, The Scripps Research Institute, La Jolla, CA, USA) with exhaustiveness (Lou et al., 2012), num modes, and energy range parameters set as 8, 30, and 10, respectively. For the docking studies the corresponding grid box parameters were used:

- PARPγ: a grid box size of 20 Å × 20 Å x 20 Å centered on–OH group of Tyr327 amino acid residue (x = 52.289, y = −30.885, z = 22.176);
- ALK5: a grid box size of 20 Å × 20 Å x 20 Å centered on–OH group of Ser280 amino acid residue (x = 2.407, y = 20.004, z = 9.543);
- p38: a grid box size of 20 Å × 20 Å x 20 Å centered on Cδ of Glu71 amino acid residue (x = 36.026, y = 31.159, z = 13.373).

Graphic visualizations of the 3D model for the poses with the lowest free energies of binding were generated using VMD 1.9 software (University of Illinois at Urbana–Champaign, Urbana, IL, USA).

#### Selected ADME parameters

ADME is the acronym for absorption, distribution, metabolism and excretion that has described pharmacokinetics for over 60 years (Nelson, 1961). ADME, as originally used, stood for descriptors quantifying drug: entering the body (A), moving about the body (D), changing within the body (M) and leaving the body (E). Over time, the use of ADME has diversified according to the needs of the user. In particular, it is used to describe mechanisms: crossing the gut wall (A); movement between compartments (D); mechanisms of metabolism (M); excretion or elimination (E); and transport (T) is sometimes added.

Selected ADME parameters (H-bond acceptors, lipophilicity, water solubility and pharmacokinetics) of the proposed compounds were theoretically calculated using available calculators, e.g., SwissADME (http://www.swissadme.ch/) (Supplementary Table S2).

Comparing the designed compounds with PFD, it can be concluded that they have parameters similar to the reference drug pirfenidone (taken orally). They meet the “rule of five” proposed by Lipinski (Lipinski, 2004): molar mass <500, logP <5, number of hydrogen bond acceptors <10.

logP–the logarithm of the ratio of the equilibrium concentrations of a given substance in *n*-octanol and water; it is a measure of the lipophilic properties of a molecule and correlates with its ability to cross biological membranes, including the blood/brain barrier, which is particularly important for drugs acting on the central nervous system. According to Lipinski’s “rule of five,” compounds with logP values below 5 (optimal value between 1 and 4) should be well absorbed after oral administration. According to Lipinski’s “rule of five,” compounds with fewer than 10 hydrogen bond acceptor sites should be well absorbed after oral administration.

### Chemical synthesis

The synthesis of 6-oxo-1-phenyl-1,6-dihydropyridine-3-carboxylic acid **4** was carried out according to the procedure (Zhang and Sommers, 2012) (Supplementary Scheme S1). Below we present the preparation of analogues with amino acids **6b** and **6f** by the mixed anhydride method.

#### Synthesis of methyl 2-(6-oxo-1-phenyl-1,6-dihydropyridine-3-carboxamido)propanoate 6b

100 mg 6-oxo-1-phenyl-1,6-dihydropyridine-3-carboxylic acid (0.46 mmol, 1 eq) was dissolved in anhydrous DMF. The mixture was cooled to −15 °C and 51 μL NMM (0.46 mmol, 1 eq) followed by 60 μL isobutyl chloroformate (0.46 mmol, 1 eq) were added. Five minutes later, the 65 mg L-alanine methyl ester hydrochloride (0.46 mmol, 1 eq) neutralized by 51 μL NMM (0.46 mmol, 1 eq) in anhydrous DMF was added into solution. The reaction mixture was stirred at −15 °C for 4 h then atroom temperature for 24 h. After evaporating the solvent *in vacuo*, the crude product was purified by thin layer chromatography (SiO_2_; 20:1/chloroform:methanol) to obtain 82 mg methyl 2-(6-oxo-1-phenyl-1,6-dihydropyridine-3-carboxamido)propanoate **6b** (0.27 mmol, 59% yield). MS-ESI *m/z* calcd C_16_H_16_N_2_O_4_ 300.31, found 301.2 [M+1]^+^; ^1^H NMR (500 MHz, DMSO-*d*_*6*_), δ (ppm): 8.61 (d, *J* = 6.7 Hz, 1H, NH), 8.36 (d, *J* = 2.4 Hz, 1H, CH), 7.96–7.90 (m, 1H, CH), 7.58–7.51 (m, 2H, Ph), 7.50–7.44 (m, 3H, Ph), 6.53 (d, *J* = 9.6 Hz, 1H, CH), 4.40 (p, *J* = 7.2 Hz, 1H, CH), 3.61 (s, 3H, COOCH_3_), 1.33 (d, *J* = 7.3 Hz, CH_3_). ^13^C NMR (500 MHz, DMSO-*d*_*6*_), δ (ppm): 173.60 (C-CO), 163.70 (C-CO), 161.52 (C-CO), 141.26 (C-Ph), 140.92 (C-C2), 139.22 (C-C4), 129.61 (C-Ph), 129.06 (C-Ph), 127.35 (C-Ph), 120.07 (C-C5), 112.40 (C-C3), 52.33 (C-CH_3_), 48.64 (C-A2), 17.13 (C-A2).

#### Synthesis of methyl 2-(6-oxo-1-phenyl-1,6-dihydropyridine-3-carboxamido)-3-phenyl propanoate 6f

100 mg 6-oxo-1-phenyl-1,6-dihydropyridine-3-carboxylic acid (0.46 mmol, 1 eq) was dissolved in anhydrous DMF. The mixture was cooled to −15 °C and 51 μL NMM (0.46 mmol, 1 eq) followed by 60 μL isobutyl chloroformate (0.46 mmol, 1 eq) were added. Five minutes later, the 99 mg L-phenylalanine methyl ester hydrochloride (0.46 mmol, 1 eq) neutralized by 51 μL NMM (0.46 mmol, 1 eq) in anhydrous DMF was added into solution. The reaction mixture was stirred at −15 °C for 4 h then at room temperature for 24 h. After evaporating the solvent *in vacuo*, the crude product was purified by thin layer chromatography (SiO_2_; 20:1/chloroform:methanol) to obtain 91 mg methyl 2-(6-oxo-1-phenyl-1,6-dihydropyridine-3-carboxamido)-3-phenylpropanoate **6f** (0.24 mmol, 52% yield). MS-ESI *m/z* calcd C_22_H_20_N_2_O_4_ 376.41, found 377.2 [M+1]^+^; ^1^H NMR (500 MHz, DMSO-*d*_6_): δ_H_8.74 (d, *J* = 7.7 Hz, 1H, NH), 8.29 (m, 1H, CH), 7.90 (dd, *J* = 9.6, 2.4 Hz, 1H, CH), 7.57 (m, 2H, Ph), 7.51 (m, 1H, Ph), 7.47 (m, 2H, Ph), 7.27 (m, 4H, Ph), 7.20 (m, 1H, Ph), 6.54 (d, *J* = 9.6 Hz, 1H), 4.65 (q, 1H, CH), 3.62 (s, 3H, COOCH_3_), 3.12 (dd, *J* = 13.8, 5.6 Hz, 1H, CH), 3.02 (dd, *J* = 13.7, 9.9 Hz, 1H). ^13^C NMR (500 MHz, DMSO-*d*_*6*_): δ_C_ 172.60 (C-CO), 163.87 (C-CO), 161.48 (C-CO), 141.33 (C-Ph), 140.91 (C-C2), 139.08 (C-C4), 137.88 (C-Ph), 129.66 (C-Ph), 129.45 (C-Ph), 129.11 (C-Ph), 128.74 (C-Ph), 127.33 (C-Ph), 127.01 (C-Ph), 120.13 (C-C5), 112.41 (C-C3), 54.62 (C-F2), 52.41 (C-CH_3_), 36.84 (C-F3).

### Biological research

#### Cell cultures

##### Fibroblasts

In presented study the immortalized human pulmonary fibroblasts purchased from abm (cat. T0490) were used. Cell line was cultured on collaged-coated t25 flasks or 24-well plates in PriGrow III medium (abm) supplemented with 1% penicillin/streptomycin solution and 10% fetal bovine serum at 4 × 10^4^ cells/cm^2^. Selected cultures media were supplemented with TGF-β1 (1 ng/mL; MERCK) and **6b** or **6f** analogues for 48 h. Since pirfenidone analogues have been dissolved in DMSO, DMSO-treated cells served as control.

##### Peripheral blood mononuclear cells

Peripheral blood mononuclear cells (PBMCs) were isolated from buffy coats derived from four volunteer donors admitted to Regional Centre for Blood Donation and Treatment in Gdańsk. The study was approved by the Ethics Committee of the Medical University of Gdańsk (NKBBN/243/2021). Our investigation was carried out in accordance with the Code of 8 Ethics of the World Medical Association (Declaration of Helsinki) for experiments on human subjects. Cells were isolated using histopaque (Sigma) gradient centrifugation. Isolated PBMCs were cultured on 24-well plates in RPMI 1640 supplemented with 10% fetal bovine serum at 1 × 10^6^/ml. During experiments cells were stimulated with anti-CD3/CD28 antibodies-coated beads for 72h and **6b** or **6f** analogues for 48 h. Since pirfenidone analogues have been dissolved in DMSO, DMSO-treated cells served as control.

#### MTT assay

The viability of fibroblasts cultured in the presence of analogues of 5-substituted-2(1*H*)-pyridone was evaluated using the MTT test. Analogues were dissolved using DMSO and than diluted in concentrations of 1ng/mL-1mg/mL. 10^4^ fibroblasts per well of 96-well plate were cultured for 24 h. After this time media were supplemented with **6b** and **6f** analogues for additional 48 h. Finally, MTT solution was added, followed by 4-h incubation. Obtained formazan crystals were dissolved with DMSO and the absorbance of the resulting solution was measured at 570 nm. The inhibition rate for blank controls was estimated as 0.

#### Proliferation test

Trypsin/EDTA (Sigma)-harvested fibroblasts or venous blood-isolated PBMCs were labeled with Violet Proliferation Dye 450 dye (VPD450; BD Biosciences) prior to creating cell cultures. For VPD450 labeling cells were washed twice in PBS and resuspended at up 3 × 10^7^ cells/mL in pre-warmed PBS with VPD450 at the final concentration of 1 μM. Cells were incubated for 20 min (fibroblasts) or 10 min (PBMCs) at 37 °C. Afterwards cells were washed with PBS and resuspended in appropriate medium with supplements. After 72 h cells were harvested and analyzed with the use of flow cytometry. VPD450 can be used to monitor cell divisions as it passively diffuses across cell membranes and is cleaved by esterases in viable cells to create highly fluorescent form. During cell divisions, the VPD450 dye is equally distributed between daughter cells, hence the reduction of fluorescence intensity, measured as Mean Fluorescence Intensity (MFI), with each cell division.

#### Flow cytometry analyses

Cultured fibroblasts were labelled with monoclonal antibodies (BD Biosciences, USA) specific for HLA-DR (cat. 560652) and CD184/CXCR4 (cat. 555976). For PBMCs 1 μL/mL of GolgySTOP (BD Biosciences) was added into wells 4 h before culture completion. Intracellular staining for TGF-β (BioLegend, USA; cat. 349610) and IL-17 (BD Biosciences, USA; 560488) was performed with ready-to-use BD Cytofix/Cytoperm Plus (BD Biosciences, USA) kit according to the manufacturer’s suggestions. The expression of cell surface (fibroblasts) and intracellular markers (PBMCs) was assessed using flow cytometry (FACSCantoII, BD Biosciences, USA, USA) after gating on live cells determined by scatter characteristics. Data was analyzed by FACSDiva 6.1.3 software (BD Biosciences, USA).

#### Scratch test

Scratch test was utilized to measure the migration of pulmonary fibroblasts treated with pyridone analogues. Cells were cultured on 24-well plates in complete PriGrow III medium until they reached ∼90% confluence. The scratch was scraped with the tip of the sterile pipette to create a cell-free zone. Cells were washed twice with warm PBS to remove detached fibroblasts and fresh medium with TGF-β and analogues was added into wells. The scratched area at the same position was monitored and recorded for 24 h to analyzed closure rate.

Statistical analysis: Statistical analyses has been performed using Statistica 13 software. ANOVA Friedman and Wilcoxon signed-rank tests have been used for multiple comparisons.

## Results

We propose the synthesis of new pirfenidone derivatives by introducing natural amino acids into the system and determining the structure-anti-fibrotic activity relationship. We propose to obtain new derivatives at position C-5 of the pyridone ring as a result of the formation of an amide bond between the carboxyl group at position 5 and the amino group of the corresponding amino acids (Supplementary Table S1). The designed compounds will be obtained by an S_N_(acyl) reaction between 5-substituted-2(1*H*)-pyridones, obtained as described in the literature (or modified) and amino acid derivatives (Lederer and Martinez, 2018; Lou et al., 2012; Mossotti et al., 2017; Nuovo et al., 2012; Parimon et al., 2020; Shi et al., 2022; Pelaia et al., 2007; Richeldi et al., 2017). Planned analogs will be synthesized in solution using various condensing reagents (e.g., mixed anhydrides, DPPA, TBTU, CDI) (Dzierzbicka et al., 2005; Kukowska-Kaszuba et al., 2011).

**SCHEME 1:**
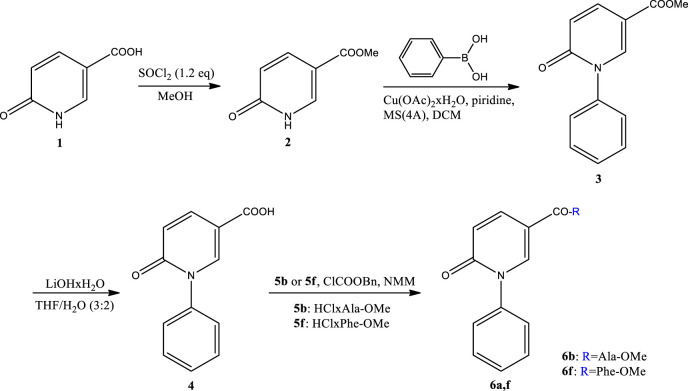
Synthesis of new derivatives at position C-5 of the pyridone ring. The numbering of compounds **6b** and **6f** is given in Supplementary Table S1.

In the first stage of the synthesis, starting from 6-hydroxynicotinic acid **1**, we obtain methyl ester 6-hydroxynicotinic acid **2**, which in reaction with phenylboronic acid, copper (II) acetate monohydrate and pyridine in DCM (dichloromethane) gives methyl ester **3**. After deprotection **3** LiOHxH_2_O in THF/water we get 5-carboxy-1-phenyl-2(*H*)-pyridinone **4** (Chen et al., 2012). In the next stage of the synthesis, compound **4** reacts with the appropriate amino acids **5** by the mixed anhydride method with or in the presence of an appropriate condensing reagent to give methyl esters **6** (Scheme 1). The structure of all compounds obtained will be confirmed on the basis of ^1^H NMR, ^13^C NMR and MS spectra. Compounds **6b** and **6f** are >99% pure by HPLC. Detailed procedures can be found in the (SI).

To examine the possible interactions of newly designed compounds within the active site of three selected molecular targets compounds were docked into the crystal structure of the PARPγ, ALK5, and p38 proteins (Protein Data Bank accession codes: 2PRG, 1RW8, and 1KV2, respectively) (Trott and Olson, 2010). Our docking experiments revealed that newly designed compounds could, at least theoretically, possess PARPγ, ALK5, and p38-binding abilities, however, the differences in the binding free energy values occurred to be significant (Supplementary Table S1).

**FIGURE 1:**
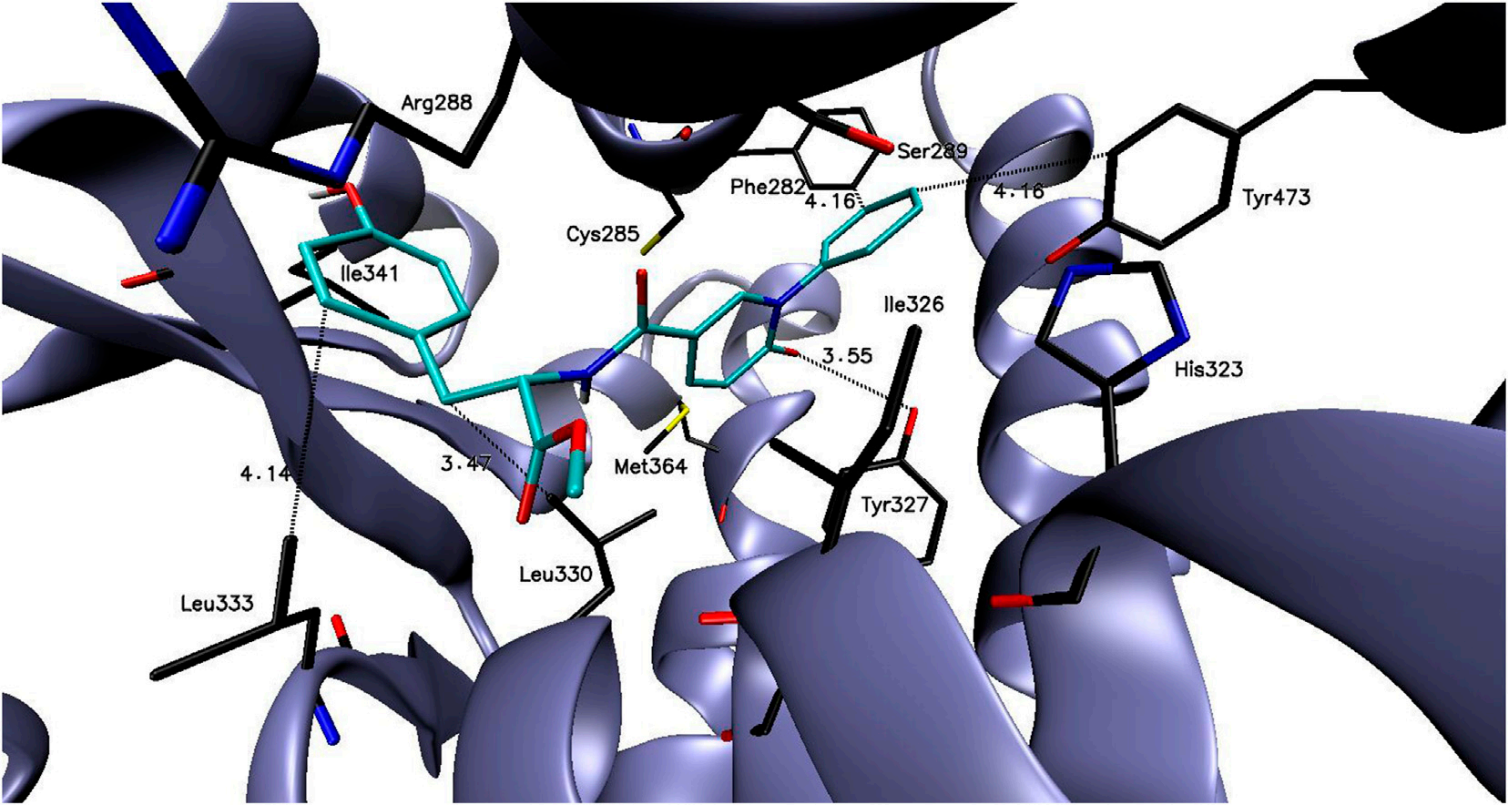
The binding mode of compound: **6k** in the PARPγ active site.

In case of docking to PARPγ protein the calculated binding free energy values of designed compounds were in the range of −5.7 to −9.2 kcal/mol (Supplementary Table S1). The most favorable binding abilities were demonstrated by compound **6k** with the binding free energy value of −9.2 kcal/mol. Importantly, the binding free energy value of compound **6k** was significantly lower than the binding free energy value of reference compound **PFD** (the binding free energy value of −6.6 kcal/mol). The molecular docking studies indicated that **6k** may create several stabilizing interaction with the amino acid residues of the PARPγ active site, which are presented in Figure 1.

**FIGURE 2:**
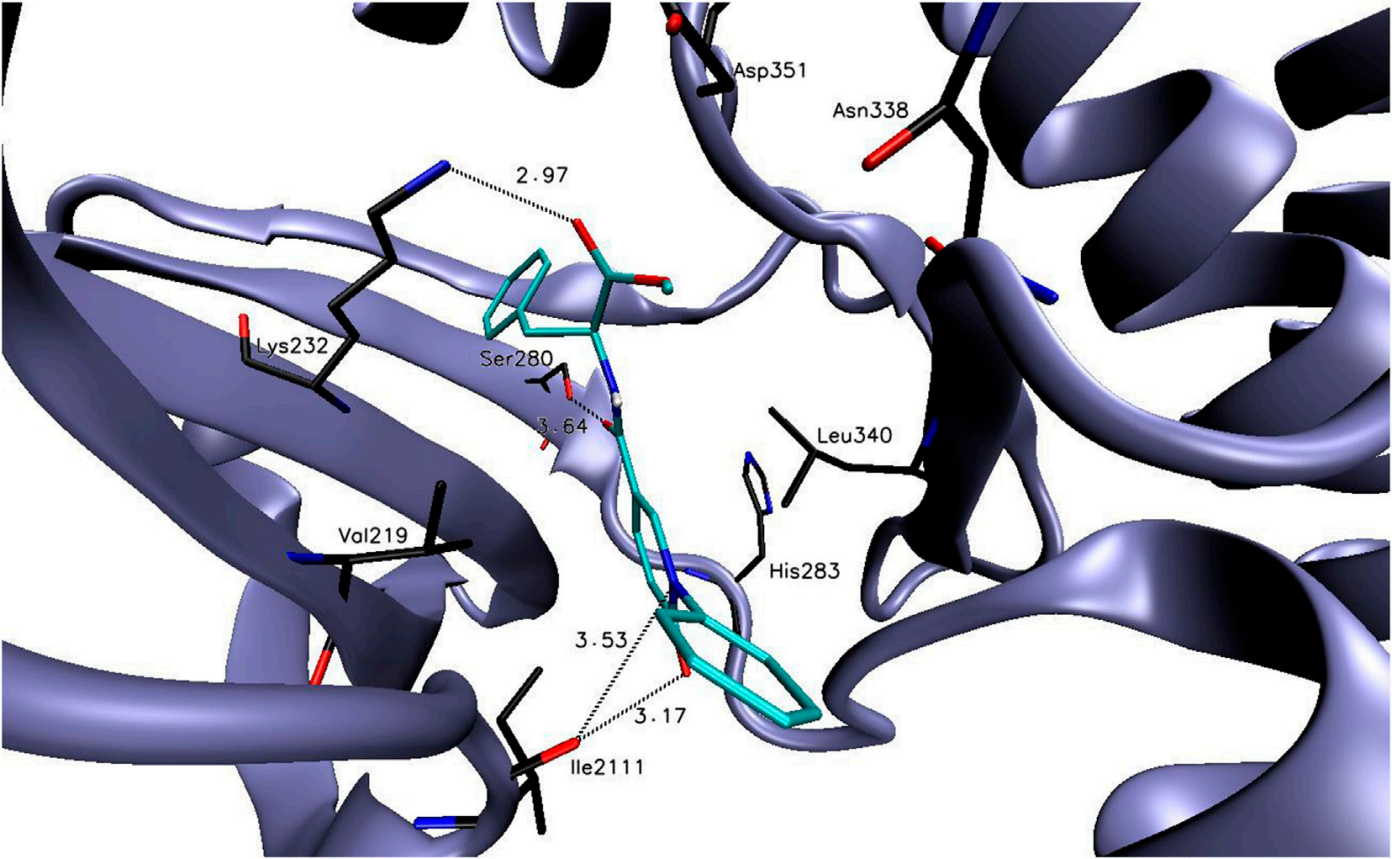
The binding mode of compound: **6f** in the ALK5 active site.

Docking studies to the ALK5 active site indicated that the binding free energy values of designed compounds were in the range of −6.1 to −9.6 kcal/mol (Supplementary Table S1). The lowest binding free energy value was calculated for compound **6f** (the binding free energy value of −9.6 kcal/mol) and it was significantly lower than for reference compound **PFD** (the binding free energy value of −7.7 kcal/mol). The putative binding mode of compound **6f** to the ALK5 active site with plausible interactions is presented in the Figure 2.

**FIGURE 3:**
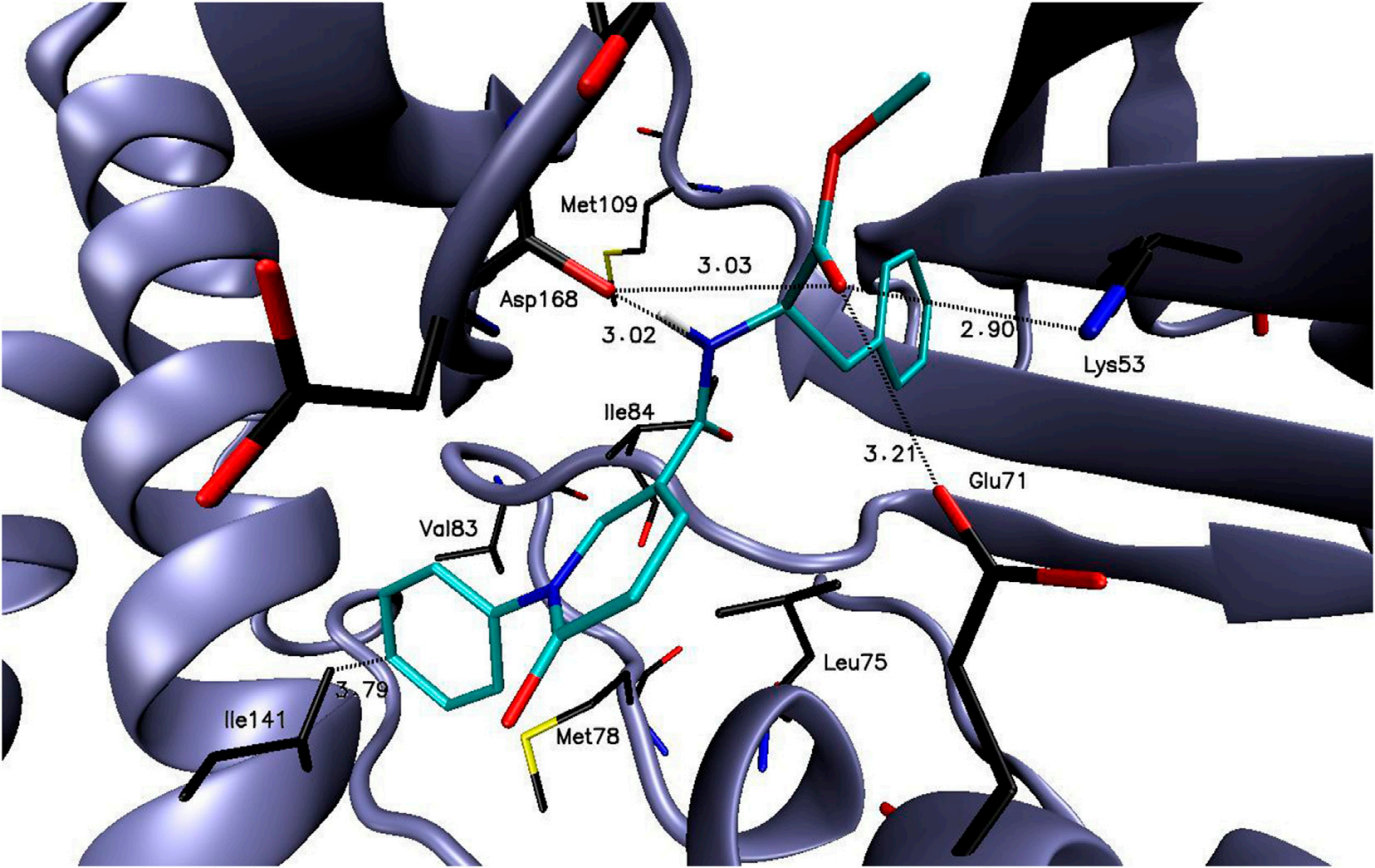
The binding mode of compound: **6m** in the p38 active site.

The binding free energy values of compounds **6a-6m** were in the range of −6.0 to −10.3 kcal/mol (the binding free energy value of reference compound **6b** was −7.4 kcal/mol, Supplementary Table S1). In the course of undertaken studies, compound **6m** showed the most favorable binding free energy value of −10.3 kcal/mol. As it is presented in the Figure 3, compound **6m** may create several electrostatic interaction with the amino acid presented the p38 active site, what may potentially stabilize the compound-enzyme complex.

In our initial research we have synthesized and performed biological *in vitro* study for two analogues selected on the basis of molecular modeling: **6b** and **6f**. Detailed biological research can be found in the SI.

### MTT test

**FIGURE 4:**
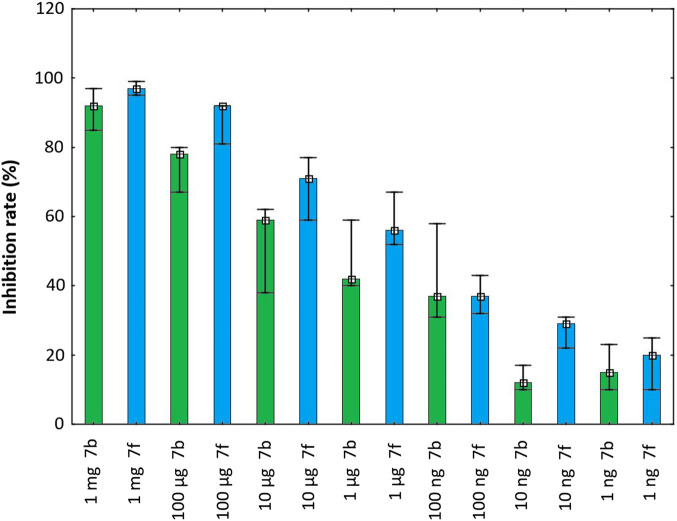
Inhibition rate of pirfenidone derivatives at different concentrations on the proliferation of pulmonary fibroblasts after 48 h.

**FIGURE 5:**
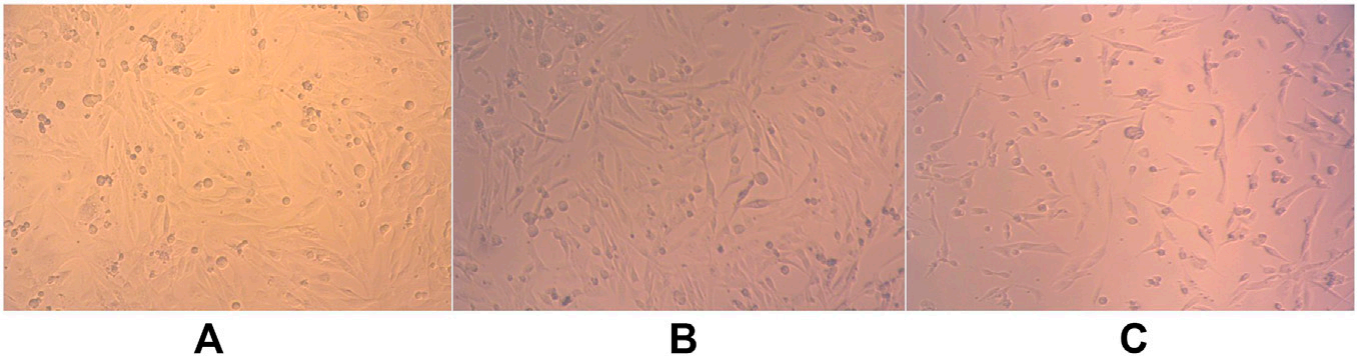
**(A–C)** Pulmonary fibroblasts after 24 h of culturing: a. control, b. **6b**, c. **6f**.

MTT test have been performed to select concentrations of PFD derivatives for subsequent analysis–according to obtained results we have chosen 1 μg/mL of **6b** (MW = 300.31) and **6f** (MW = 376.41). Results of MTT test are shown in Figure 4. Figures 5A–C present fibroblasts after 24 h of culturing–photographs indicate that **6f** exhibited greater potential in blocking cell proliferation when compared to control or **6b**–stimulated cells.

### Flow cytometry analysis

**FIGURE 6:**
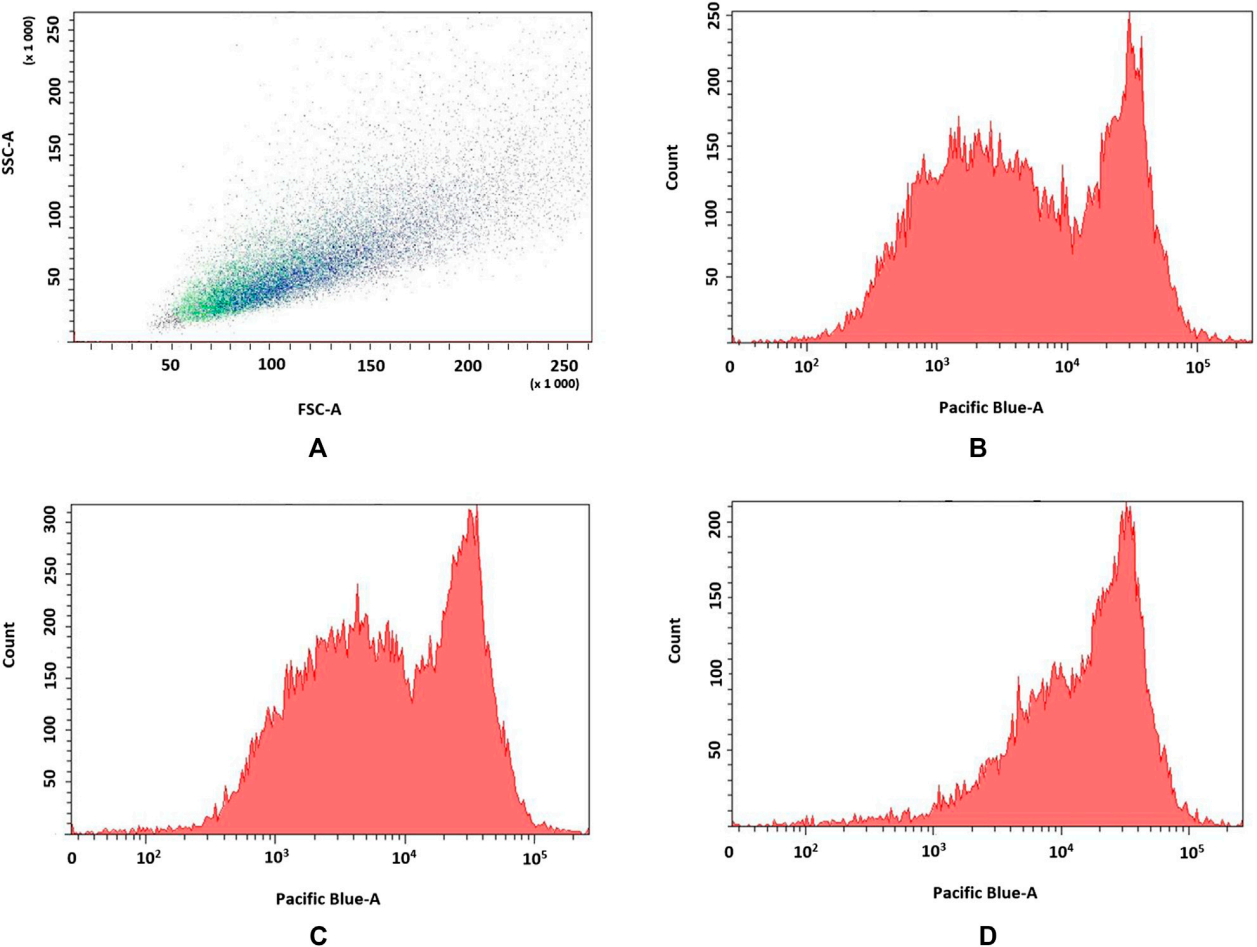
**(A–D)** Flow cytometry analyses of TGF-β1-stimulated fibroblasts. a. FSC vs. SSC dot plot of fibroblasts, b. control; c. **6b**, d. **6f**.

Effect of pirfenidone derivatives on pulmonary fibroblasts were confirmed by flow cytometry analyses of 72 h proliferation by TGF-β1-stimulated fibroblasts (n = 3). **6f** derivative more efficiently inhibited cell proliferation (MFI = 22.350 ± 402) when compared to control (MFI = 13.456 ± 311) and **6b**–stimulated (MFI = 15.886 ± 412) cells as indicated by higher VPD450 fluorescence (p = 0.049) (Figures 6A–D).

We have analyzed HLA-DR and CXCR4 expression on fibroblasts, but have not observed significant differences in the percentages of HLA-DR- or CXCR4-positive cells as well as fluorescence intensities (data not shown).

**FIGURE 7:**
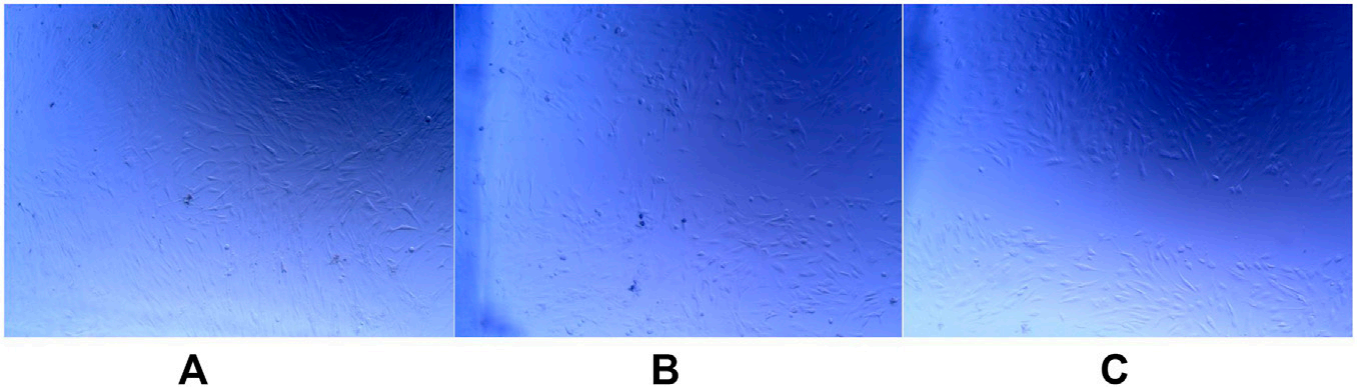
**(A–C)** Effect of pyridine analogues on fibroblasts migration after 24 h culture; a. control, b. **6b**, c. **6f**.

We have also analyzed 24 h migration of TGF-β1-stimulated fibroblasts (n = 4) with data being calculated as wound closure after 24 h in comparison to wound area at 0 h. Performed analyses revealed that **6f** derivative (19.7% ± 8.4%) inhibited migration more efficiently, when compared to **6b** (31.2% ± 10.14%) or control cells (96.4% ± 4.7) (p = 0.02) (Figures 7A–C).

**FIGURE 8:**
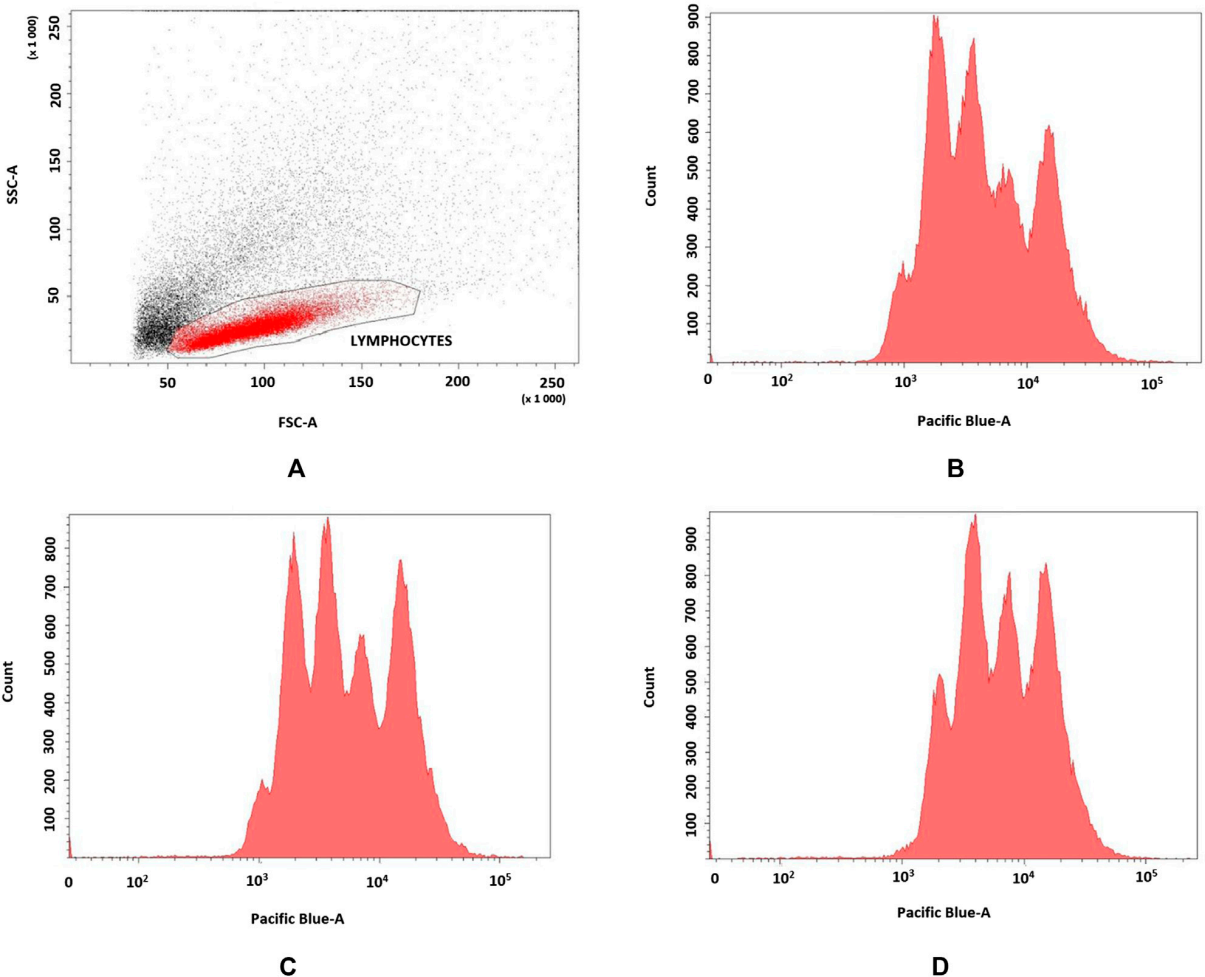
**(A–D)** Effect of pyridine analogues on beads-stimulated PBMCs proliferation; a. FSC vs. SSC dot plot of PBMCs, b. control; c. **6b**, d. **6f**.

We have also analyzed effect of pyridine analogues on blood mononuclear cells (PBMCs) (n = 3). The results of 72 h proliferation assay indicated that leukocytes less frequently underwent divisions in the presence of **6f** (MFI = 10.201 ± 396) when contrasted with control (MFI = 7.509 ± 204) or **6b** analogue (MFI = 8.858 ± 453) (p = 0.05) (Figures 8A–D).

**FIGURE 9:**
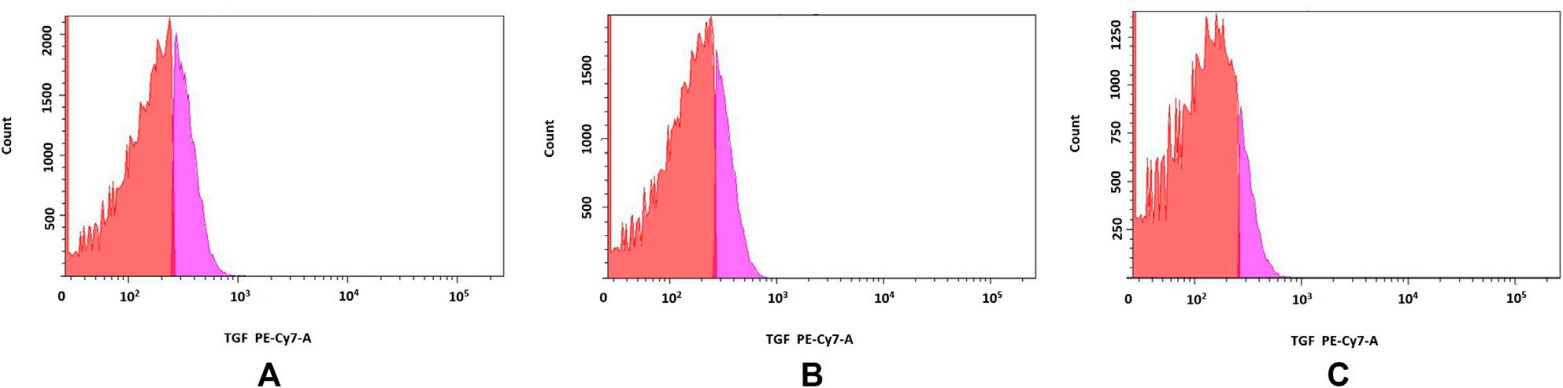
**(A–C)** TGF-β1 production by PBMCs. Red part of histogram indicates TGF-β1-negative cells; pink part of histogram distinguishes TGF-β1-producing cells. a. control, b. **6b**, c. **6f**.

PFD derivatives also affected TGF-β1 production by beads-stimulated PBMCs (n = 3) – fewer leukocytes produced cytokine in the presence of **6f** (8.1% ± 0.8%) when compared to control (26.8% ± 2.1%) and **6b**–stimulated (20.4% ± 5.3%) cells (p = 0.049) (Figures 9A–C).

**FIGURE 10:**
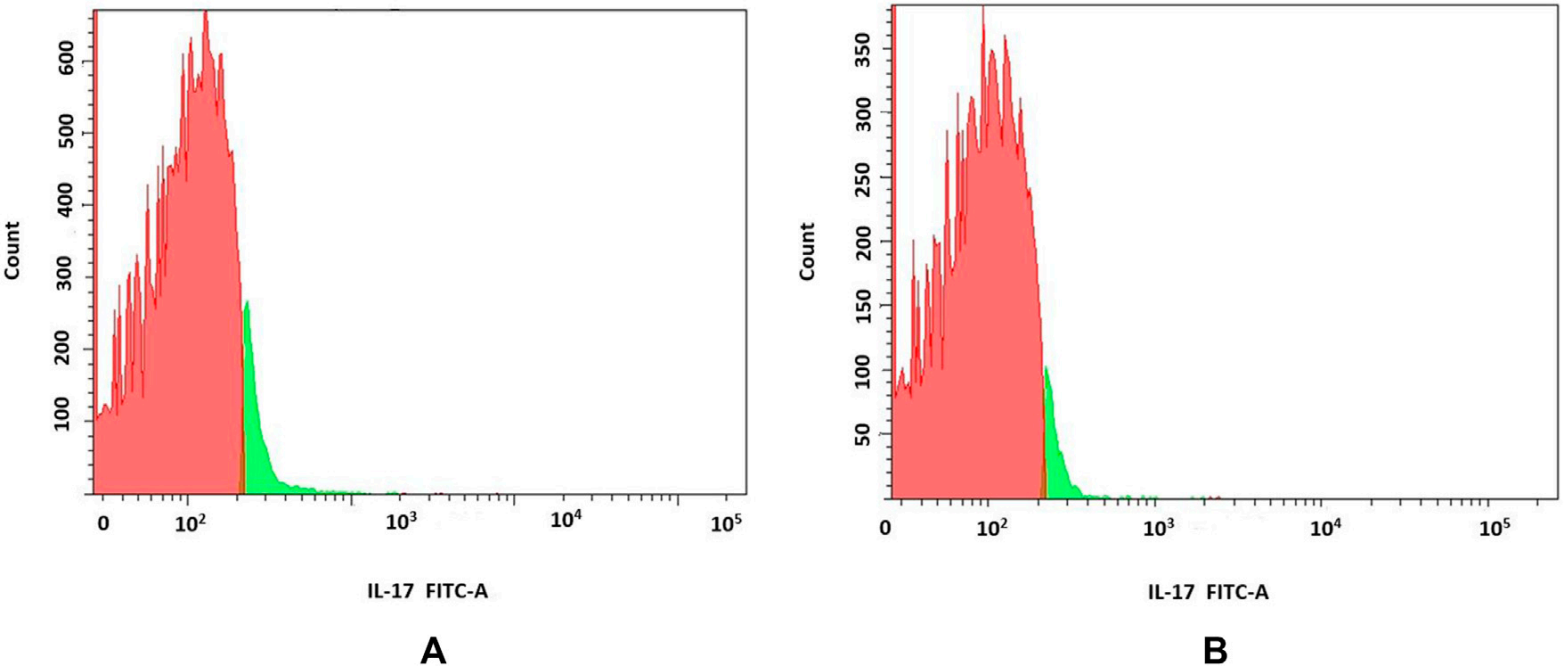
**(A,B)** Analyses of IL-17 production by PBMCs; Red part of histogram indicates IL-17-negative cells; green part of histogram indicates IL-17-producing cells a. control, b. **6f**.

We have also explored IL-17 production by beads-stimulated PBMCs (n = 2). In the presence of **6f** 3% ± 0.9% cells produced IL-17 when compared to 6.1% ± 1.4% of control cells. However, the differences did not reach statistical significance (p = 0.18) (Figures 10A,B).

## Discussion

We report, for the first time, the evaluation *in silico* of series of pirfenidone analogues combined with L-amino acids derivatives as a potential drugs in IPF. Preliminary *in vitro* studies of two derivatives show that they are worth further extensive testing. We have shown that distinguished analogues affect proliferation of pulmonary fibroblasts with the **6f** compound exhibiting more potent effect. Moreover, both compounds severely impacted migration of these cells, but did not alter the expression of HLA-DR and CXCR4 molecules. Similarly, PFD derivatives inhibited proliferation of activated PBMCs and decreased the production of TGF-β1 and IL-17.

TGF-β is described as main pro-fibrotic cytokine that stimulates: proliferation, recruitment, secretory activity and differentiation of fibroblasts (Sakai and Tager, 2013). Cytokine also inhibits proliferation (Ryan et al., 1994) and induces apoptosis in AECs (Solovyan and Keski-Oja, 2006), therefore its involvement in IPF may be also connected with causing loss of epithelial cells and disturbing their proper regeneration (Fang et al., 2020). Overall, TGF-β is recognized as the most relevant cytokine in pulmonary fibrosis. Protein is known to be highly expressed in fibrotic lungs with various cells being indicated as its producers, including: AECs, fibroblasts, myofibroblasts, neutrophils and alveolar macrophages (Sakai and Tager, 2013; Fang et al., 2020). Its administration in animal models is known to induce myofibroblasts accumulation and fibrosis, whereas TGF-β blockage suppresses those processes (Sakai and Tager, 2013).

IL-17 is highly inflammatory cytokine that induces production of various subsequent cytokines and chemokines, such as granulocyte macrophage-colony stimulating factor (GM-CSF), tumor necrosis factor or IL-1β (Ito et al., 2015). It also affects migration and differentiation of lung neutrophils (Aulakh, 2018) as well as contributes to airway tissue remodeling by inducing production of profibrotic cytokines in fibroblasts (Pelaia et al., 2007). Regions of active fibrosis are characterized by increased expressiof numerous proteins, including IL-17 when compared to normal lung (Nuovo et al., 2012). This cytokine is also increased in bronchoalveolar lavage (BAL) fluids isolated from IPF sufferers. Celada et al. described abundance of proliferative-deficient, TGF-β and IL-17 co-expressing CD4^+^ cells in IPF patients that induce collagen synthesis in fibroblasts (Celada et al., 2018). Others also confirmed that lung fibroblasts proliferate, differentiate into myofibroblasts and synthesize extracellular matrix components when IL-17 stimulated (Zhang et al., 2019). Similar, promising results concerning different pirfenidone derivatives has been published by Yao et al. (2023).

We believe that conducted experiments constitute preliminary studies evaluating pirfenidone analogs with promising antifibrotic properties.

## Conclusion

In this manuscript, we have presented preliminary results of studies concerning two compounds from a designed series of 5-substituted-2(1*H*)-pyridone derivatives with L-amino acids to investigate their potential as idiopathic pulmonary fibrosis (IPF) therapeutics. We have conducted molecular docking studies against PARPg, ALK5, and p38, in order to prove that compounds **6f**, **6k**, and **6m** interacted more strongly with their receptors when compared to standard pirfenidone (PFD). We have also synthesized two compounds and performed preliminary *in vitro* analyzes using human lung fibroblasts and PBMCs confirming our hypothesis. Compound **6f** outperformed **6b** in inhibiting fibroblast proliferation and migration and suppressing IL-17 and TGF-β1 production in PBMCs. The presented preliminary research results are so interesting that in the future we will analyze a larger number of derivatives, taking into account the PFD model in the research.

## Data Availability

The original contributions presented in the study are included in the article/Supplementary Material, further inquiries can be directed to the corresponding author.
