# Supplementary material for: New analogs of 5-substituted-2(1H)-pyridone containing of natural amino acids as potential drugs in idiopathic pulmonary fibrosis. Investigation in silico and preliminary in vitro
Source: Acta Biochim Pol. 2025 Nov 10;72:15218. doi: 10.3389/abp.2025.15218 (PMC12640879; doi:10.3389/abp.2025.15218)
Supplement: Supplementary file 1 [file DataSheet1.docx]

New analogs of 5-substituted-2(1*H*)-pyridone containing of natural amino acids as potential drugs in idiopathic pulmonary fibrosis. Investigation *in silico* and preliminary *in vitro*

Krystyna Dzierzbicka^1*^, Maria Skrzypkowska^2^, Monika Gensicka-Kowalewska^1^, Mateusz Daśko^3^ and Bartosz Słomiński^2^

*^1^Department of Organic Chemistry, Gdansk University of Technology, Gdansk, Poland,*

*^2^Department of Medical Immunology, Medical University of Gdansk, Gdansk, Poland,*

*^3^Department of Inorganic Chemistry, Gdansk University of Technology, Gdansk, Poland,*

*Correspondence:*

*Krystyna Dzierzbicka*

*krydzier@pg.edu.pl*

KEYWORDS: *Pirfenidone, PFD, Amino acids, Idiopathic Pulmonary Fibrosis, IPF*

**Computational studies**

**Ligands preparation for molecular docking**

The 3D structure of ligands **6a-6m** (**Table 1**) were prepared with the Portable HyperChem 8.0.7 Release (Hypercube, Inc., Gainesville, FL, USA). Prior to docking calculations, the structure of each ligand was optimized using a MM+ force field and the Polak–Ribière conjugate gradient algorithm (terminating at a gradient of 0.05 kcal mol^−1^ Å^−1^).

**Protein preparation for molecular docking**

The X-ray structures of the PARPγ, ALK5, and p38 used for molecular modeling studies were taken from the Protein Databank (Protein Data Bank accession codes: 2PRG, 1RW8, and 1KV2, respectively). After standard preparation procedure (including removal of water molecules and other ligands as well as addition hydrogen atoms and Gasteiger charges to each atom) docking analysis was carried out. In the case of PARPγ, the amino acid chains B and C were additionally removed from the structure 2PRG.

**Molecular docking**

Docking studies were carried out using Autodock Vina 1.1.2 software (The Molecular Graphic Laboratory, The Scripps Research Institute, La Jolla, CA, USA) with exhaustiveness,^16^ num modes, and energy range parameters set as 8, 30, and 10, respectively. For the docking studies the corresponding grid box parameters were used:

- PARPγ: a grid box size of 20 Å x 20 Å x 20 Å centered on –OH group of Tyr327 amino acid residue (x = 52.289, y = -30.885, z = 22.176);

- ALK5: a grid box size of 20 Å x 20 Å x 20 Å centered on –OH group of Ser280 amino acid residue (x = 2.407, y = 20.004, z = 9.543);

- p38: a grid box size of 20 Å x 20 Å x 20 Å centered on Cδ of Glu71 amino acid residue (x = 36.026, y = 31.159, z = 13.373).

Graphic visualizations of the 3D model for the poses with the lowest free energies of binding were generated using VMD 1.9 software (University of Illinois at Urbana–Champaign, Urbana, IL, USA).

**Table 1.** The binding free energy values calculated for potential PARPγ, ALK5, and p38 inhibitors **6a-6m** and reference compound PFD.

| **No.** | **Structure** | **Binding free Energy (kcal mol^-1^)** | | |
| --- | --- | --- | --- | --- |
|  |  | **PPARγ** | **ALK5** | **p38** |
| **PFD** |  | -6.6 | -7.7 | -7.4 |
| **6a** |  | -8.1 | -8.4 | -8.2 |
| **6b** |  | -8.2 | -8.2 | -8.4 |
| **6c** |  | -8.3 | -8.7 | -7.9 |
| **6d** |  | -8.5 | -8.8 | -9.4 |
| **6e** |  | -8.2 | -8.8 | -9.0 |
| **6f** |  | -8.9 | -9.6 | -9.8 |
| **6g** |  | -8.7 | -9.4 | -7.9 |
| **6h** |  | -8.4 | -9.1 | -8.7 |
| **6i** |  | -8.6 | -9.0 | -8.4 |
| **6j** |  | -8.0 | -8.2 | -8.3 |
| **6k** |  | -9.2 | -9.3 | -9.9 |
| **6l** |  | -8.0 | -8.6 | -8.8 |
| **6m** |  | -8.5 | -9.4 | -10.3 |

ADME can theoretically be calculated using available calculators, e.g. SwissADME (<http://www.swissadme.ch/>) (**Table 2**).

**Table 2.** Selected ADME parameters (H-bond acceptors, lipophilicity, water solubility and pharmacokinetics) of the proposed compounds.

| **No.** | **Num. H-bond acceptors** |  | | |
| --- | --- | --- | --- | --- |
|  |  | **LogPo/w (iLOGP)** | **LogS (ESOL)** | **GI absorption** |
| **PFD** | 1 | 2.34 | -2.73  3.46e-01 mg/ml; 1.87e-03 mol/l | High |
| **6a** | 4 | 2.70 | -2.18  1.88e+00 mg/ml;  6.55e-03 mol/l | High |
| **6b** | 4 | 2.63 | -2.51  9.29e-01 mg/ml;  3.09e-03 mol/l | High |
| **6c** | 4 | 2.92 | -3.19  2.13e-01 mg/ml;  6.48e-04 mol/l | High |
| **6d** | 4 | 3.21 | -3.42  1.30e-01 mg/ml;  3.79e-04 mol/l | High |
| **6e** | 4 | 3.47 | -3.42  1.30e-01 mg/ml;  3.79e-04 mol/l | High |
| **6f** | 4 | 3.15 | -3.92  4.56e-02 mg/ml;  1.21e-04 mol/l | High |
| **6g** | 4 | 2.85 | -2.89  4.18e-01 mg/ml;  1.28e-03 mol/l | High |
| **6h** | 5 | 1.88 | -2.10  3.08e+00 mg/ml;  7.98e-03 mol/l | High |
| **6i** | 5 | 1.80 | -2.70  7.34e-01 mg/ml;  2.00e-03 mol/l | High |
| **6j** | 4 | 3.01 | -3.04  3.29e-01 mg/ml;  9.14e-04 mol/l | High |
| **6k** | 5 | 3.04 | -3.78  6.52e-02 mg/ml;  1.66e-04 mol/l | High |
| **6l** | 5 | 2.20 | -2.21  2.06e+00 mg/ml;  6.23e-03 mol/l | High |
| **6m** | 4 | 3.15 | -3.92  4.56e-02 mg/ml;  1.21e-04 mol/l | High |

Lipophilicity - LogPo/w(iLOGP); Water solubility - LogS (ESOL); Pharmacokinetics - GI (Gastrointestinal) absorption

**Chemical Synthesis**

The synthesis of 6-oxo-1-phenyl-1,6-dihydropyridine-3-carboxylic acid **4** was carried out according to the procedure (Zhang and Sommers 2012), and compounds **6b** and **6f** by the mixed anhydride method (**Scheme 1**).

Synthesis of methyl-6-oxo-1,6-dihydropyridine-3-carboxylate **2**

To a mixture of 4.00 g 6-oxo-1,6-dihydropyridine-3-carboxylic acid (28.75 mmol, 1 eq) **1** in 10 ml anhydrous methanol at 0⁰C was carefully added 2.52 ml SOCl_2_ (34,53 mmol, 1.2 eq). After being refluxed for 6 h, the reaction was cooled down to rt and the solvent was evaporated to dryness to give 3 g methyl-6-oxo-1,6-dihydropyridinr-3-carboxylate (19.59 mmol, 68% yield) as a brown powder; melting point 166-172⁰C.

Synthesis of methyl 6-oxo-1-phenyl-1,6-dihydropyridine-3-carboxylate **3**

A mixture of 3 g methyl-6-oxo-1,6-dihydropyridinr-3-carboxylate (19.59 mmol, 1 eq), 2.87 g phenylboronic acid (23.53 mmol, 1.2 eq), 5.88 g copper(II) acetate monohydrate (29.41 mmol, 1.5 eq), 3.16 ml pyridine (39.16 mmol, 2 eq) and 3 g molecular sieves (4 Å) in 50 ml dichloromethane were stirred on a magnetic stirrer for 12 hours at ambient temperature. The organic layer was washed with H_2_O, dried (MgSO_4_), filtered and concentrated under reduced pressure. Further purification by column chromatography (SiO_2_; 0.02:1/methanol:chloroform) gave 2.5 g methyl 6-oxo-1-phenyl-1,6-dihydropyridine-3-carboxylate (10.91 mmol, 56% yield) as yellow powder; melting point 100-105⁰C.

Synthesis of 6-oxo-1-phenyl-1,6-dihydropyridine-3-carboxylic acid **4**

To a mixture of 1 g methyl 6-oxo-1-phenyl-1,6-dihydropyridine-3-carboxylate (4.37 mmol, 1 eq) in 9 ml THF and 6 ml H_2_O at 0⁰C was added 0.37 g LiOHxH_2_O (8.74 mmol, 2 eq). After one hour of stirring on a magnetic stirrer, the solution was diluted with water and washed twice with ethyl acetate. Then the water layer was acidified with 2N HCl to pH 2. The white powder obtained was filtered off. 0.74 g of 6-oxo-1-phenyl-1,6-dihydropyridine-3-carboxylic acid (3.44 mmol, 79% yeld) with a melting point of 256-263⁰C was obtained.

**Methyl 2-(6-oxo-1-phenyl-1,6-dihydropyridine-3-carboxamido)propanoate 6b**


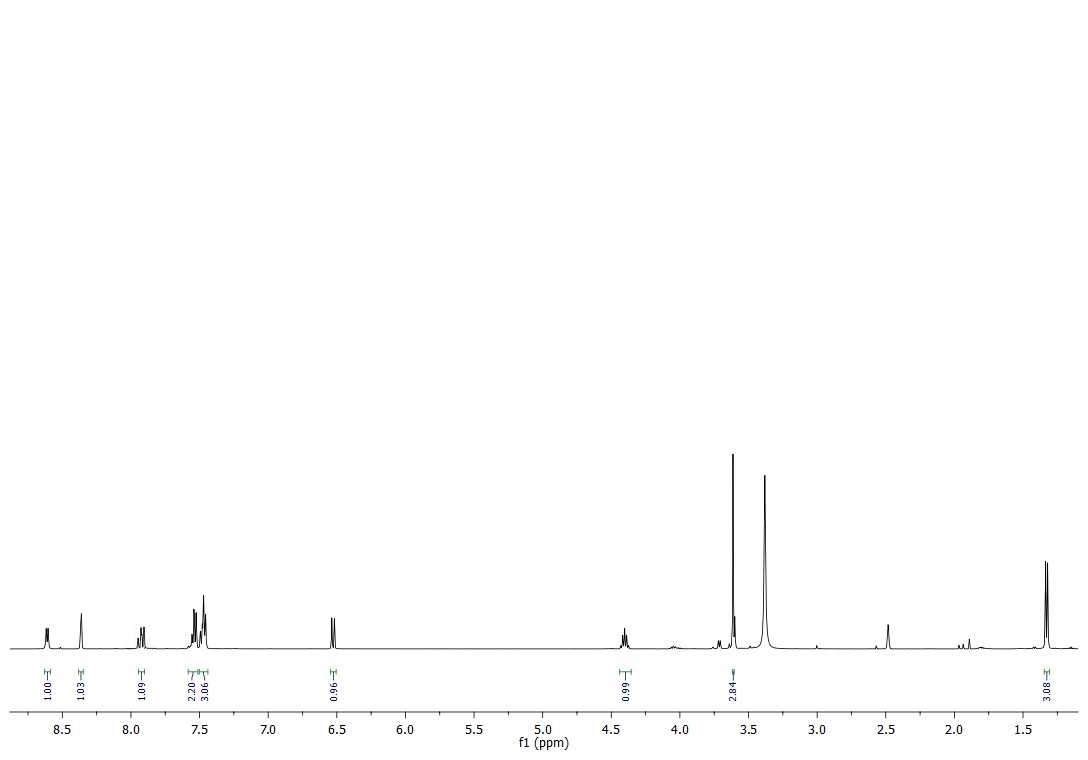


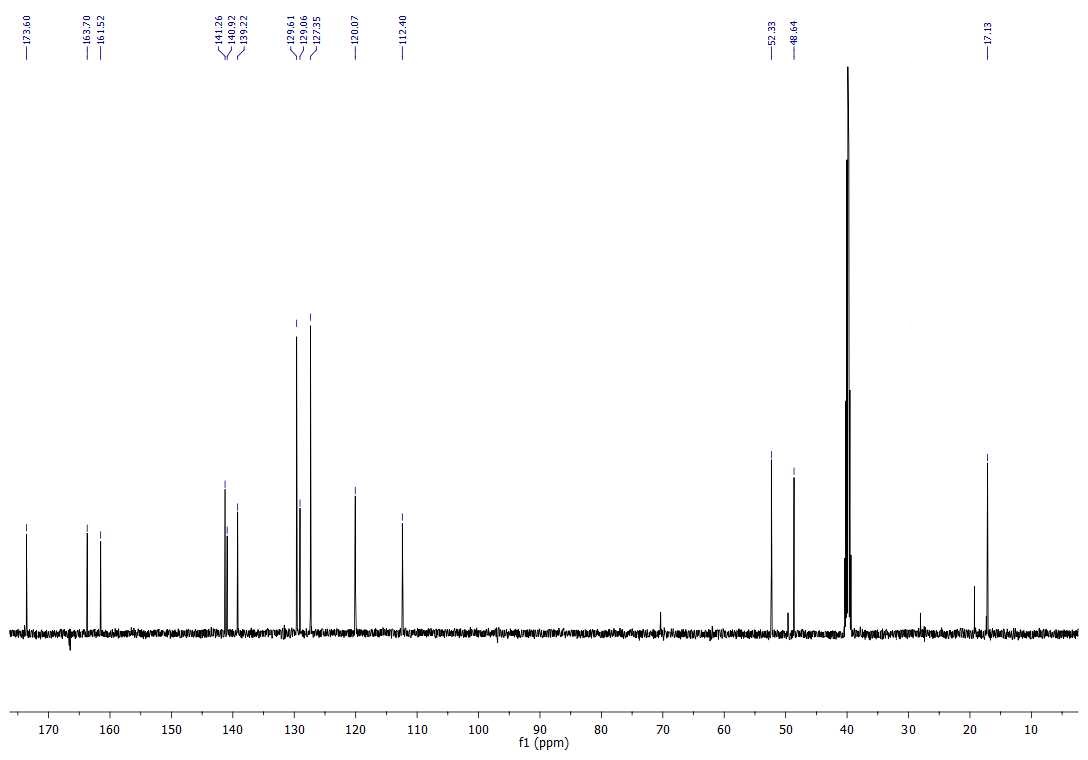


**Methyl 2-(6-oxo-1-phenyl-1,6-dihydropyridine-3-carboxamido)-3-phenylpropanoate 6f**


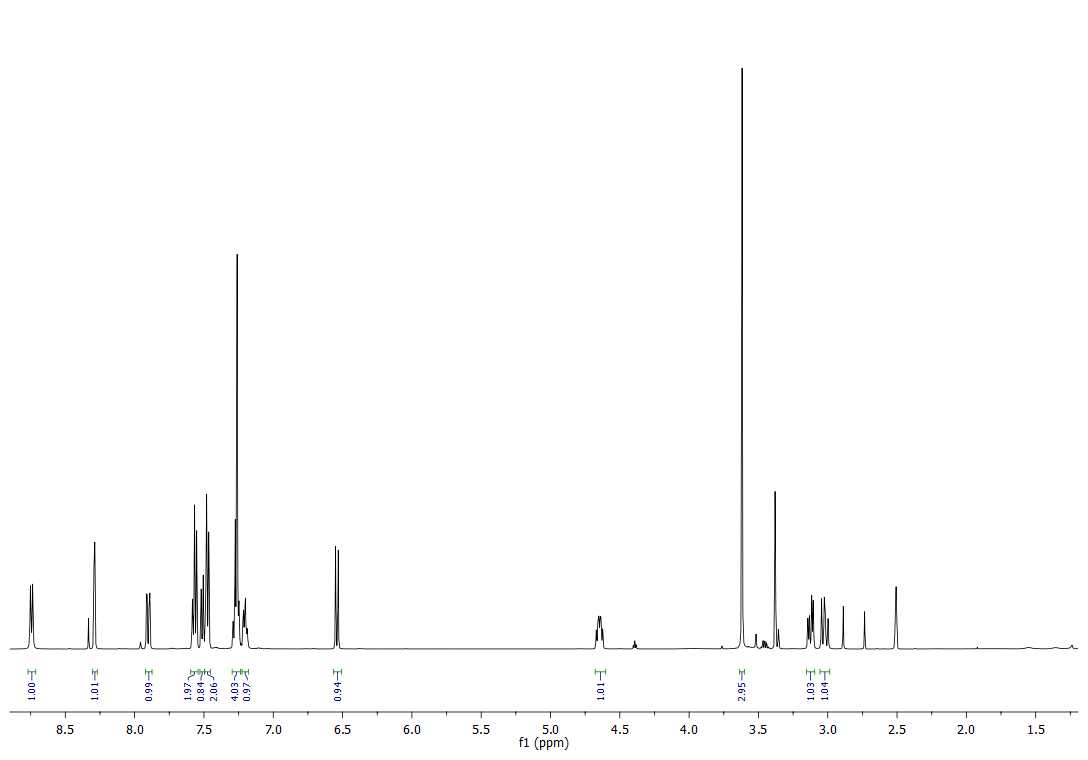


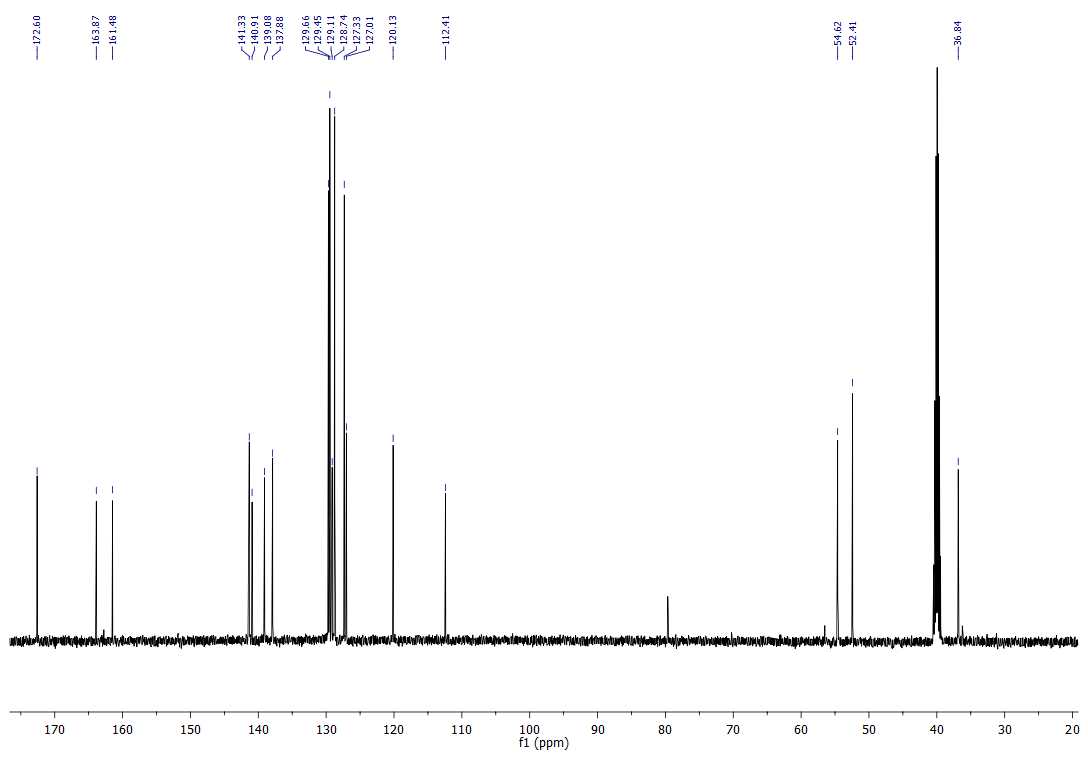


1. Equipment

Chromatographic analysis was performed using Agilent liquid chromatograph series 1290 (Agilent Technology, Waldbronn, Germany) containing: high speed binary pump (G7120A), autosampler (G7167B), thermostated column compartment (G7116B), and diode-array detector (G1315C). The Agilent MassHunter software controlled the chromatographic system.

2. *Chromatographic analysis*

Samples were dissolved in DMSO and injected (2 µL) onto an RP-C18 silica gel column (Poroshell EC; 2.7 µm, 3.0 × 150 mm, Agilent Technologies) thermostated at 40 °C. The mobile phase flow rate was 0.4 mL min⁻¹, and elution was carried out using 0.1% (v/v) formic acid in water (solvent A) and ACN/MeOH (1:1, v/v) (solvent B). The gradient elution program was as follows: 0 min, 20% B; 0–15 min, linear increase to 100% B; 15–25 min, 100% B. The analysis was stopped after 25 min. The column was re-equilibrated for 15 min at 20% B. The UV signal was monitored at 254, 280, and 350 nm.

Compound **6b**

min

2.5

5

7.5

10

12.5

15

17.5

20

22.5

mAU

0

200

400

600

800

1000

1200

1400

6.085

8.206

Purity: 99.93 %

mAU

0

200

400

600

800

1000

1400

1600

UV spectra of compound **6b**

minnm

2.5250

5300

7.5350

10400

12.5450

15500

17.5550

20

22.5

mAU

0

250

500

7501200

1000

1250

1500

1750

2000

DAD1 A, Sig=254,8 Ref=off (KN_Phe) Compound **7f**

11.787

15.760

Purity: 99.96 %

nm

250

300

350

400

450

500

550

mAU

0

500

1000

1500

2000

UV spectra of compound **6f**

**Biological Research**

**Cell cultures**

**Fibroblasts**

In presented study the immortalized human pulmonary fibroblasts purchased from abm (cat. T0490) were used. Cell line was cultured on collaged-coated t25 flasks or 24-well plates in Prirow III medium (abm) supplemented with 1% penicillin/streptomycin solution and 10 % fetal bovine serum at 4x10^4^ cells/cm^2^. Selected cultures media were supplemented with TGF-β1 (1 ng/ml; MERCK) and **6b** or **6f** analogues for 48h.

**Peripheral blood mononuclear cells**

Peripheral blood mononuclear cells (PBMCs) were isolated from buffy coats derived from four volunteer donors admitted to Regional Centre for Blood Donation and Treatment in Gdańsk. Cells were isolated using histopaque (Sigma) gradient centrifugation. Isolated PBMCs were cultured on 24-well plates in RPMI 1640 supplemented with 10 % fetal bovine serum at 1x10^6^/ml. Cells were stimulated with anti-CD3/CD28 antibodies-coated beads for 72h and **6b** or **6f** analogues for 48h.

**MTT assay**

The viability of fibroblasts cultured in the presence of analogues of 5-substituted-2(1*H*)-pyridone was evaluated using the MTT test. Analogues were dissolved using DMSO and than diluted in concentrations of 1ng/ml-1mg/ml. 10^4^ fibroblasts per well of 96-well plate were cultured for 24h. After this time media were supplemented with **6b** and **6f** analogues for additional 48h. Finally, MTT solution was added, followed by 4-h incubation. Obtained formazan crystals were dissolved with DMSO and the absorbance of the resulting solution was measured at 570 nm.

# Proliferation test

Trypsin/EDTA (Sigma)-harvested fibroblasts or venous blood-isolated PBMCs were labeled with Violet Proliferation Dye 450 dye (VPD450; BD Biosciences) prior to creating cell cultures. For VPD450 labeling cells were washed twice in PBS and resuspended at up 3x10^7^ cells/ml in pre-warmed PBS with VPD450 at the final concentration of 1 μM. Cells were incubated for 20 minutes (fibroblasts) or 10 minutes (PBMCs) at 37°C. Afterwards cells were washed with PBS and resuspended in appropriate medium with supplements. After 72h cells were harvested and analyzed with the use of flow cytometry. VPD450 can be used to monitor cell divisions as it passively diffuses across cell membranes and is cleaved by esterases in viable cells to create highly fluorescent form. During cell divisions, the VPD450 dye is equally distributed between daughter cells, hence the reduction of fluorescence intensity, measured as Mean Fluorescence Intensity (MFI), with each cell division.

**Flow cytometry analyses**

Cultured fibroblasts were labelled with monoclonal antibodies (BD Biosciences, USA) specific for HLA-DR (cat. 560652) and CD184/CXCR4 (cat. 555976). For PBMCs 1 µl/ml of GolgySTOP (BD Biosciences) was added into wells 4 hours before culture completion. Intracellular staining for TGF-β (BioLegend, USA; cat. 349610) and IL-17 (BD Biosciences, USA; 560488) was performed with ready-to-use BD Cytofix/Cytoperm Plus (BD Biosciences, USA) kit according to the manufacturer’s suggestions. The expression of cell surface (fibroblasts) and intracellular markers (PBMCs) was assessed using flow cytometry (FACSCantoII, BD Biosciences, USA, USA) after gating on live cells determined by scatter characteristics. Data was analyzed by FACSDiva 6.1.3 software (BD Biosciences, USA).

**Scratch test**

Scratch test was utilized to measure the migration of pulmonary fibroblasts treated with pyridone analogues. Cells were cultured on 24-well plates in complete PriGrom III medium until they reached ~90 % confluence. The scratch was scraped with the tip of the sterile pipette to create a cell-free zone. Cells were washed twice with warm PBS to remove detached fibroblasts and fresh medium with TGF-β and analogues was added into wells. The scratched area at the same position was monitored and recorded for 24h to analyzed closure rate.

**Statistical analysis:** Statistical analyses will be performed using Statistica 13 software. ANOVA Friedman and Kruskal-Wallis test will be used for multiple comparisons.
